# Cigarette smoke induced urocystic epithelial mesenchymal transition via MAPK pathways

**DOI:** 10.18632/oncotarget.14456

**Published:** 2017-01-02

**Authors:** Dexin Yu, Hao Geng, Zhiqi Liu, Li Zhao, Zhaofeng Liang, Zhiqiang Zhang, Dongdong Xie, Yi Wang, Tao Zhang, Jie Min, Caiyun Zhong

**Affiliations:** ^1^ Department of Urology, The Second Affiliated Hospital of Anhui Medical University, Hefei 230032, China; ^2^ Department of Preventive Medicine and Public Health Laboratory Sciences, School of Medicine, Jiangsu University, Jiangsu 212013, China; ^3^ Department of Toxicology and Nutritional Science, School of Public Health, Nanjing Medical University, Nanjing 210029, China

**Keywords:** bladder cancer, cigarette smoke, epithelial mesenchymal transition, MAPK pathways

## Abstract

Cigarette smoke has been shown to be a major risk factor for bladder cancer. Epithelial-mesenchymal transition (EMT) is a crucial process in cancer development. The role of MAPK pathways in regulating cigarette smoke-triggered urocystic EMT remains to be elucidated. Human normal urothelial cells and BALB/c mice were used as *in vitro* and *in vivo* cigarette smoke exposure models. Exposure of human normal urothelial cells to cigarette smoke induced morphological change, enhanced migratory and invasive capacities, reduced epithelial marker expression and increased mesenchymal marker expression, along with the activation of MAPK pathways. Moreover, we revealed that ERK1/2 and p38 inhibitors, but rather JNK inhibitor, effectively attenuated cigarette smoke-induced urocystic EMT. Importantly, the regulatory function of ERK1/2 and p38 pathways in cigarette smoke-triggered urocystic EMT was further confirmed in mice exposed to CS for 12 weeks. These findings could provide new insight into the molecular mechanisms of cigarette smoke-associated bladder cancer development as well as its potential intervention.

## INTRODUCTION

Bladder cancer is the fifth most common cancer in Western countries and the most common cancer in urinary tract [1]. In 2013 there were estimated 382,700 new cases of bladder cancer worldwide, with 143,000 resultant deaths all over the world [2]. Cigarette smoking (CS) has been proven as the major risk factors for bladder cancer [3, 4]. It is estimated that CS causes-almost half of male patients and a quarter of female patients of bladder cancer in the United State [5]. Although enormous progresses have been made in understanding the molecular mechanisms leading to bladder cancer development, the tumorigenic process is still poorly understood.

Epithelial-to-mesenchymal transition (EMT), a process characterized by loss of homotypic cadhesion and cell polarity and increased invasion and migration, plays essential roles in development and wound healing [6]. Numerous *in vitro* and *in vivo* studies suggest that EMT is associated with cancer cell invasion and metastasis in various malignancies, including bladder cancer [6, 7]. EMT is also a critical player in the early stage of tumorigenesis [8–11]. CS-induced EMT has been found to regulate early events in carcinogenesis [12]. Yet the mechanisms regarding how CS induces EMT remain to be elucidated.

The mitogen-activated protein kinases (MAPKs) belong to a family of serine/threonine kinases that play central roles in tumorigenic process [12]. MAPK pathways not only promotes cell proliferation, differentiation and survival, but also mediates oncogenesis and is upregulated in cancer cells [13, 14]. Compelling evidence demonstrates that MAPK/AP-1 activity is critical for the effects of CS [15, 16]. Recently, some groups reported that ERK1/2, JNKs, and p38 regulate EMT [17–20]. However, few studies have been focused on MAPK regulation of CS-induced urocystic EMT. Although we previously found that curcumin inhibited CS-induced EMT and MAPK activation in the bladder of mice [12], and that ERK5 promoted CS-induced urocystic EMT *in vitro* [21], the function of ERK1/2, P38 and JNK MAPK pathways in CS-associated urocystic EMT remains unknown.

The present study aimed to examine the role of ERK1/2, p38 and JNK pathways in CS-elicited EMT in both normal urothelial cells and bladder tissues. Findings from this study could provide important information for the molecular mechanisms of CS-related bladder tumorigenesis.

## RESULTS

### CSE elicited EMT in normal urothelial cells

**Figure 1:**
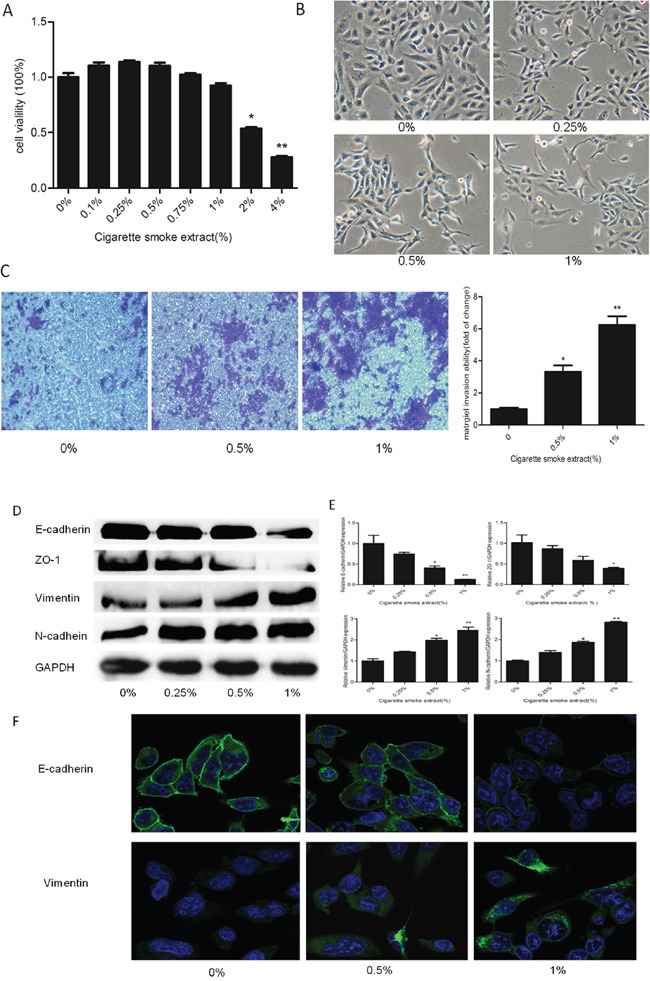
CSE induced EMT in SV-HUC-1 cells **A**. MTT assay showed cell viability decreased below 80% when cells were exposed to 2% or higher CSE concentrations in SV-HUC-1 cells. **B**. CSE induced morphological change from epithelial to spindle-like mesenchymal shape. SV-HUC-1 cells became longer and thinner, some of which generated slender tails. **C**. Transwell invasion assay revealed CS made a strong stimulative effect on the invasion capacity of SV-HUC-1 cells. The subsequent absorbance assay confirmed this change. **D**. CSE decreased the expression of epithelial markers E-cadherin and ZO-1, and increased expression of mesenchymal markers Vimentin and N-cadherin in SV-HUC-1 cells by Western blotting. **E**. CSE decreased the expression of E-cadherin and ZO-1 mRNAs, and enhanced the expression of Vimentin and N-cadherin mRNAs, detected by qRT-PCR. Data are expressed as mean ± SD. *p < 0.05, ** p < 0.01, compared with control group. **F**. Immunofluorescent staining also showed that CSE decreased E-cadherin protein expression and increased Vimentin expression in SV-HUC-1 cells.

Following the treatment of human SV-HUC-1 cells with various concentrations of CSE for 5 days, the cell viability was determined by MTT assay. The results showed that 2% or higher concentrations of CSE were cytotoxic to SV-HUC-1 cells since the cell viability was significantly reduced when compared with the control group (Figure 1A). Consequently, we chose 1% CSE as the highest CSE concentration for the subsequentexperiments.

The EMT process is characterized by alterations of cell morphology, migrative and invasive capacity, as well as epithelial and mesenchymal markers’ expression. CSE treatment for 5 days led to significant morphological change of SV-HUC-1 cells, i.e., from a urothelial oblate-shape to a spindle-like mesenchymal form (Figure 1B). To examine the alterations of EMT markers, Western blot and qRT-PCR were carried out. We found that the protein levels of epithelial markers E-cadherin and ZO-1 were significantly decreased by CSE treatment. On the contrary, CSE treatment significantly increased the expression levels of mesenchymal proteins Vimentin and N-cadherin (Figure 1D). Similar changes were observed for the mRNA expression of epithelial and mesenchymal markers in CSE-treated SV-HUC-1 cells (Figure 1E). Likewise, immunofluorescence staining confirmed that CSE reduced E-cadherin expression and elevated Vimentin expression (Figure 1F). Futhermore, transwell assays revealed that CSE enhanced the invasion of SV-HUC-1 cells through reconstituted matrigel matrices(Figure 1C). Together, these results demonstrated that CSE elicited EMT in normal urothelial cells.

### CSE-triggered urocystic EMT was associated with activation of MAPK pathways

**Figure 2:**
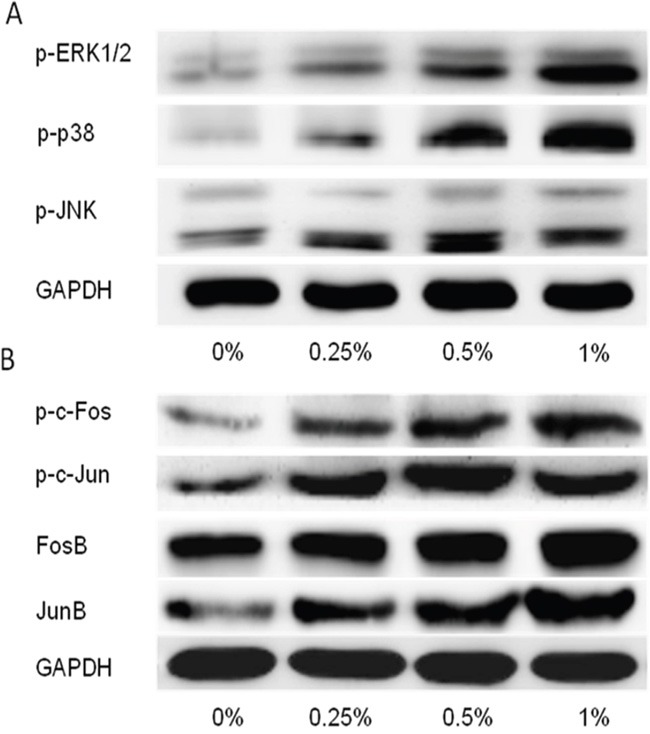
CS–induced EMT is associated with activation of MAPK/AP-1 activation in SV-HUC-1 cells **A**. CSE activated ERK1/2, p38, JNK activation in SV-HUC-1 cells following CSE treatment for 5 days in protein level. **B**. CSE increased AP-1 proteins activation following CSE treatment for 5 days. GAPDH was used as loading control.

The activation status of MAPK pathways was determined in SV-HUC-1 cells following CSE treatment for 5 days. It was shown that CSE remarkably activated the level of phosphorylated ERK1/2, p38 and JNK in the cells in a dose-dependent manner (Figure 2A). Moreover, CSE provoked the activation of AP-1 proteins in SV-HUC-1 cells, as evidenced by increased levels of AP-1 components (p-c-Jun, p-c-Fos, FosB and JunB) (Figure 2B).

### Inhibitors of ERK1/2 and p38 attenuated CSE-triggered urocystic EMT

**Figure 3:**
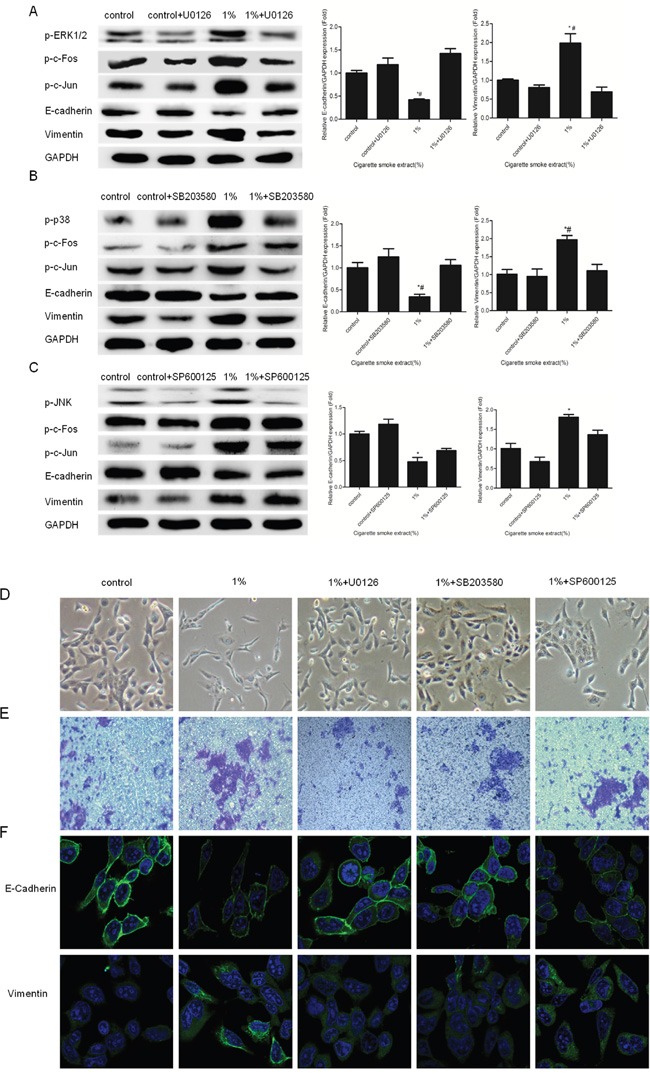
Inhibition of ERK1/2 and p38 attenuated CS-induced EMT in SV-HUC-1 cells **A**. U0126 suppressed the activation of p-ERK1/2, p-c-Fos, p-c-Jun and alterations of EMT markers induced by CS exposure. **B**. SB203580 diminished the activation of p-p38, p-c-Fos, p-c-Jun and alterations of EMT markers induced by CS exposure. **C**. SP600125 could not decreased the activation of p-c-Fos and alterations of EMT markers induced by CS exposure. **D**. SV-HUC-1 cells became collective and full after treated with U0126 and SB203580. SP600125 can not attenuated morphological change triggered by CS. **E**. Enhanced invasion capacity of SV-HUC-1 cells triggered by CSE was reversed by U0126 and SB203580. **F**. Immunofluorescent staining confirmed U0126 and SB203580 not only increased the expression of E-cadherin in membrane but also decreased the expression of Vimentin in cytoplasm. * p< 0.05 and ** p < 0.01, represented CSE group compared with control group. # p<0.05, represented inhibitor of MAPK pathways group compared with respective CSE group.

Next, we examined the role of MAPKs in CSE-induced EMT in SV-HUC-1 cells. ERK1/2 inhibitors (U0126, 5μM), JNK inhibitors (SP600125, 2μM), and p38 inhibitors (SB203580, 5μM) were used according to previous reports [22–24]. Our results revealed that ERK1/2 and p38 inhibitor diminished CSE-triggered EMT in these cells. Inhibition of ERK1/2 and p38 significantly suppressed the activation of p-c-fos and p-c-jun (Figure 3A-3B). ERK1/2 and p38 inhibitor abolished CS-elicited changes in the expression of EMT markers, as measured by Western blot, qRT-PCR, and immunofluorescent assays (Figure 3A and 3F). Meanwhile, we also found U0126 and SB203580 treatment reversed CS-triggered mensenchymal-like morphological change in the cells (Figure 3D). Moreover, transwell assay also showed that U0126 and SB203580 suppressed the invasive capacity of the cells triggered by CSE. (Figure 3E). On the contrary, inhibition of JNK by SP600125 had little effect on urocystic EMT triggered by CS (Figure 3C-3F).

### CS altered EMT markers’ expression and activated MAPKs in mouse bladder tissue

**Figure 4:**
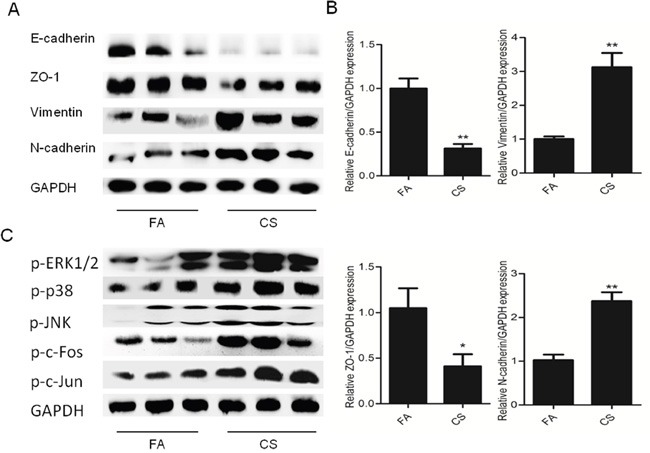
CS induced alterations of EMT markers in the bladder of mice exposed to CS for 12 weeks **A**. CS decreased the protein levels of E-cadherin and ZO-1, and increased the protein levels of Vimentin and N-cadherin in the bladder of mice. **B**. CS reduced mRNA levels of E-cadherin and ZO-1, and increased the protein levels of Vimentin and N-cadherin in the bladder of mice. **C**. CS increased ERK1/2, p38 and JNK activation in the bladder of mice. Data are expressed as mean ±SD. * p< 0.05 and ** p < 0.01, compared with FA control. FA = filtered air; CS = cigarette smoke.

Following the illustration of the function of ERK1/2 and p38 in CS-induced urocystic EMT *in vitro*, we further investigated whether CS induced EMT-like changes and modulated MAPK activation in a mouse exposure model. We exposed mice to CS for 12 conseutive weeks and the expression levels of epithelial and mesenchymal markers were determined in mouse bladder tissues. We found that CS significantly reduced the protein levels of E-cadherin and ZO-1, and increased Vimentin and N-cadherin proteins in the bladder tissues of mouse (Figure 4A). Silimar changes in mRNA expression of EMT markers was showed by qRT-PCR assays (Figure 4B). In addition, we revealed that exposure of mice to CS resulted in significant increased ERK1/2, p38 and JNK activity and increased c-Jun and c-Fos activation in bladder tissues (Figure 4C). These data were in line with our *in vitro* findings.

### Inhibitors of MAPK pathways diminished CS-induced EMT change in mouse bladder tissue

**Figure 5:**
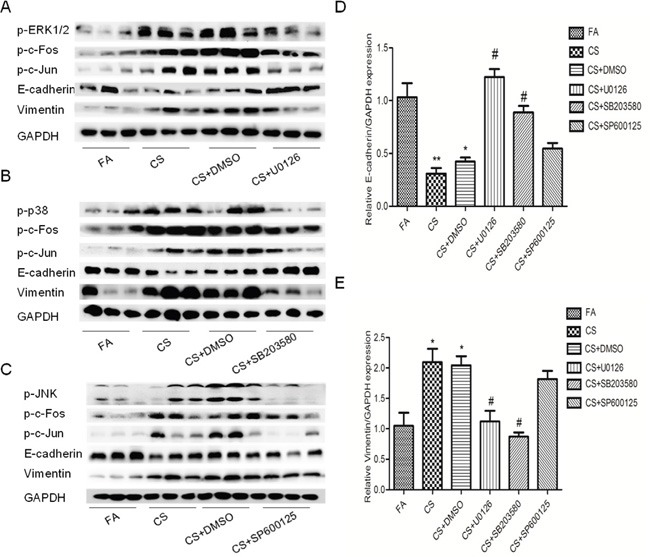
Inhibitors of ERK1/2 and p38 pathways diminished CS-induced urocystic EMT in the bladder of mice **A**. U0126 attenuated CS-induced alterations in the expression of p-c-Fos, p-c-Jun, E-cadherin and Vimentin in proteins level. **B**. SB203580 attenuated CS-induced alterations in the expression of p-c-fos, p-c-jun as well as E-cadherin and Vimentin in proteins level. **C**. SP600125 could not reverse the expression change of p-c-Fos together with E-cadherin and Vimentin in proteins level. **D**. and **E**. U0126 and SB203580 diminished CS-induced urocystic EMT in the bladder of mice in mRNA level. SP600125 could not reversed the EMT change triggered by CS. Data are expressed as mean ±SD. * p< 0.05 and ** p < 0.01, compared with FA control; # p <0.05, represented CS group compared with respective inhibitor of MAPK pathways.

To explore the role of MAPK pathway in CS-mediated EMT-like change in the bladder, mice were received MAPK inhibitors and exposed to CS for 12 conseutive weeks. Safety and toxicity examination, including body weight, diet consumption, hematology and blood biochemistry, showed that no obvious adverse effects were observed in mice treated with MAPK inhibitors when compared with the control group mice (data not shown). We showed that EKR1/2 and p38 inhibitors significantly attenuated p-c-Jun and p-c-Fos expression levels in the bladder after exposure to CS for 12 weeks (Figure 5A-5B). Moreover, our results revealed that CS-induced decrease of E-cadherin protein and increase of Vimentin protein were effectively supressed by inhibitor of ERK1/2 and p38. Similar results were observed in mRNA level (Figure 5D-5E). These data indicated that inhibitors of ERK1/2 and p38 reversed CS-induced urocystic EMT *in vivo*. However, we found that JNK inhibitor SP600125 failed to attenuate CS-eliciated EMT change in the bladder of mouse (Figure 5C-5E). These results were in accordance with our *in vitro* findings.

## DISCUSSION

By using *in vitro* and *in vivo* models, we demonstrated in the present study that CS induced urocystic EMT. We further showed that CS-triggered EMT was associated with activation of MAPK pathways. Moreover, our data indicated that inhibitors of ERK1/2 and p38, but not JNK inhibitor, effectively diminished CS-induced urocystic EMT *in vitro* and *in vivo*. Our findings suggested the critical role of ERK1/2 and p38 activity in CS-associated urocystic EMT and the underlying mechanism of CS-associated bladder cancer development.

EMT is a crucial process in cancer development [12]. In line with previous reports, we revealed that CS exposure induced EMT in human urocystic SV-HUC-1 cells, as evidenced by cellular morphological change, increased invasive ability, and altered EMT markers expression, including reduced epithelial markers E-cadherin and ZO-1, and elevated mesenchymal markers Vimentin and N-cadherin. Moreover, similar alterations were found in the bladder of mice, suggestive of EMT induction. Together, these results illustrated that CS triggered urocystic EMT in both *in vitro* and *in vivo* settings.

The regulation of EMT involves several critical cellular signalings. However, the potential mechanisms of CS-induced EMT remain unclear. Evidences have shown that ERK1/2, JNK and p38 pathways faciliate EMT [17–20, 25, 26]. Nevertheless, little information is available regarding MAPK regulation of urocystic EMT induced by CS. We demonstrated in our study that CS triggerd urocystic EMT mainly through ERK1/2 and p38 pathways, while JNK pathway exhibited little role in regulating CS-induced urocystic EMT. Since ERK1/2 is a major determinant in controlling cell growth, survival and invasion, and inhibitors of ERK1/2 have been applicated for anticancer drugs [27–29]. It has been reported that butein can inhibit the migrative and invasive capacities of human bladder cancer cells through ERK1/2 suppression [30]. Sunitinib is effective in bladder cancer patients, which suppresses the activation of ERK1/2 [31]. Moreover, evidence revealed that ERK1/2 and p38 play a role in CCDC34-mediated bladder cancer cell proliferation and migration [32]. Resistance to chemotherapeutic drugs is one of the major challenges in the treatment of cancer. Inhibition of p38 significantly increased Gemcitabine sensitivity in bladder cancer cells [33]. Fang, Y et al reported that JNK inhibitor SP600125 suppressed anchorage-independent growth of T24T bladder cancer cells [34]. In the present study we illustrated that ERK1/2 and p38 inhibitors significantly attenuated CS-induced urocystic EMT *in vitro* and *in vivo*, whereas JNK inhibitor failed to exhibit similar effects. Thus, our data suggested the differential riole of MAPK pathways in regulating CS-triggered urocystic EMT.

In summary, our study demonstrated that ERK1/2 and p38 promote CS-induced urocystic EMT in both human urothelial cells and mouse bladder tissue. These novel findings indicate the critical function of ERK1/2 and p38 activity in CS-related bladder cancer development and provide a plausible strategy for the search of potential interventional target of CS-associated bladder oncogenesis.

## MATERIALS AND METHODS

*See* the supplemental material for further details concerning methods.

### Chemicals and reagents

An SV-40 immortalized human urothelial cell line (SV-HUC-1) was purchased from Chinese Academy of Typical Culture Collection Cell Bank. F12K medium was purchased from Gibco (New York, NY, USA). Fetal bovine serum (FBS) was obtained from PAA Laboratories (Pasching, Austria). 3-(4,5-Dimethylthiazol-2-yl)-2,5-diphenyltetrazolium bromide (MTT) was purchased from Sigma-Aldrich. SB203580, SP600125, and U0126 were purchased from Beyotime (Shanghai, China). The primary antibodies for phosphorylated JNK, phosphorylated p38, phosphorylated ERK1/2, phosphorylated c-Jun, phosphorylated c-Fos, E-cadherin, ZO-1, N-cadherin and Vimentin were obtained from Cell Signaling Technology (Beverly, MA). GAPDH antibody was from Biogot Technology (Nanjing, China). Primers for E-cadherin, ZO-1, Vimentin, N-cadherin and GAPDH were synthesized by Invitrogen (Carlsbad, CA). Sources of other materials are noted accordingly in the text.

### Cell culture and treatment

SV-HUC-1 cells were cultured in F12K medium containing antibiotics (100 U/ml penicillin and 100 μg/ml streptomycin). CS extract (CSE) was freshly prepared for each experiment by combusting one filterless 3R4F reference cigarette according to reported method [35, 36]. SV-HUC-1 cells were treated with various concentrations of CSE for 5 days.

### Cell toxicity assay

MTT assay was used to detect SV-HUC-1 cells viability and appropriate CSE concentrations were chosen for further use.

### Mice and CS exposure

Eight-week-old male BALB/c mice weighing 18-22 g were purchased from the Animal Research Center of Nanjing Medical University. Mice were exposed to tobacco smoke in a smoking apparatus. The smoke was delivered to whole-body exposure chambers with target concentration of total particulate matter (TPM) of 85 mg/m^3^. Animals were exposed for 6 hours daily for 12 consecutive weeks. After the last CS exposure, mice were sacrificed and the bladder tissues were isolated, frozen and stored at -80°C until analysis.

### Treatment of mice by inhibitors of MAPK pathways

In a separate set of animal study, mice were treated daily with different MAPK inhibitors as previously reported [37–40]. All the inhibitors (0.5 mg/kg for U0126, 1 mg/kg for SB203580 and 1 mg/kg for SP600125) were dissolved in DMSO and intraperitoneally injected to the mice. Mice were randomly assigned into six groups (n=10 per group): filtered air group; CS-exposed group; CS+DMSO group; CS+U0126 group; CS+SB203580 group; CS+SP600125 group; Following the completion of exposure, mice were sacrificed and bladder tissues were collected for analysis.

### Western blot analysis

Proteins were extracted from SV-HUC-1 cells and mouse bladder tissues. Western blot analyses were performed for the determination of protein expression levels.

### Quantitative real-time PCR

Total RNA was isolated by RNAiso Plus according to the manufacturer's instructions (TaKaRa, Japan). qRT-PCR was performed using Power SYBR Green Master Mix (TaKaRa, Japan) and an ABI 7300 real-time PCR detection system (Applied Biosystems). Fold changes in expression of each gene were calculated by a comparative threshold cycle (Ct) method using the formula 2^− (ΔΔCt)^.

### Transwell assay

Invasive capacities of CSE-treated SV-HUC-1 cells were evaluated using Transwell chambers with matrigel.

### Immunofluorescence

Immunofluorescent staining was performed to analyze the expression of E-cadherin and Vimentin in SV-HUC-1 cells treated with CSE.

### Statistical analysis

Statistical analyses were performed with SPSS 16.0. All data were expressed as mean ± standard deviation. One-way ANOVA was used for comparison of statistical differences among multiple groups, followed by the LSD significant difference test. Unpaired Student t test was also used for the comparison between two groups. A value of *p<* 0.05 was considered significantly different.

## SUPPLEMENTARY MATERIALS METHODS



## Abbreviations

BC: bladder cancer
CS: cigarette smoke
CSE: cigarette smoke extract
EMT: epithelial mensenchymal transition
p-ERK1/2: phosphorylated ERK1/2
p-p38: phosphorylated p38
p-JNK: phosphorylated JNK
GAPDH: glyceraldehyde-3-; phosphate-dehydrogenase
PCR: polymerase chain reaction

## References

[1] Shen CH, Shee JJ, Wu JY, Lin YW, Wu JD, Liu YW (2010). Combretastatin A-4 inhibits cell growth and metastasis in bladder cancer cells and retards tumour growth in a murine orthotopic bladder tumour model. British journal of pharmacology.

[2] Siegel RL, Miller KD, Jemal A (2015). Cancer statistics, 2015. CA: a cancer journal for clinicians.

[3] Volanis D, Kadiyska T, Galanis A, Delakas D, Logotheti S, Zoumpourlis V (2010). Environmental factors and genetic susceptibility promote urinary bladder cancer. Toxicology letters.

[4] Boffetta P (2008). Tobacco smoking and risk of bladder cancer. Scandinavian journal of urology and nephrology Supplementum.

[5] Ploeg M, Aben KK, Kiemeney LA (2009). The present and future burden of urinary bladder cancer in the world. World journal of urology.

[6] McConkey DJ, Choi W, Marquis L, Martin F, Williams MB, Shah J, Svatek R, Das A, Adam L, Kamat A, Siefker-Radtke A, Dinney C (2009). Role of epithelial-to-mesenchymal transition (EMT) in drug sensitivity and metastasis in bladder cancer. Cancer metastasis reviews.

[7] Hanze J, Henrici M, Hegele A, Hofmann R, Olbert PJ (2013). Epithelial mesenchymal transition status is associated with anti-cancer responses towards receptor tyrosine-kinase inhibition by dovitinib in human bladder cancer cells. BMC cancer.

[8] Sun JL, Chen DL, Hu ZQ, Xu YZ, Fang HS, Wang XY, Kan L, Wang SY (2014). Arsenite promotes intestinal tumor cell proliferation and invasion by stimulating epithelial-to-mesenchymal transition. Cancer biology & therapy.

[9] Xu W, Ji J, Xu Y, Liu Y, Shi L, Liu Y, Lu X, Zhao Y, Luo F, Wang B, Jiang R, Zhang J, Liu Q (2015). MicroRNA-191, by promoting the EMT and increasing CSC-like properties, is involved in neoplastic and metastatic properties of transformed human bronchial epithelial cells. Molecular carcinogenesis.

[10] Tellez CS, Juri DE, Do K, Bernauer AM, Thomas CL, Damiani LA, Tessema M, Leng S, Belinsky SA (2011). EMT and stem cell-like properties associated with miR-205 and miR-200 epigenetic silencing are early manifestations during carcinogen-induced transformation of human lung epithelial cells. Cancer research.

[11] Liu Y, Luo F, Xu Y, Wang B, Zhao Y, Xu W, Shi L, Lu X, Liu Q (2015). Epithelial-mesenchymal transition and cancer stem cells, mediated by a long non-coding RNA, HOTAIR, are involved in cell malignant transformation induced by cigarette smoke extract. Toxicology and applied pharmacology.

[12] Liang Z, Xie W, Wu R, Geng H, Zhao L, Xie C, Li X, Zhu M, Zhu W, Zhu J, Huang C, Ma X, Wu J, Geng S, Zhong C, Han H (2015). Inhibition of tobacco smoke-induced bladder MAPK activation and epithelial-mesenchymal transition in mice by curcumin. International journal of clinical and experimental pathology.

[13] Yang SH, Sharrocks AD, Whitmarsh AJ (2013). MAP kinase signalling cascades and transcriptional regulation. Gene.

[14] Sangrar W, Shi C, Mullins G, LeBrun D, Ingalls B, Greer PA (2014). Amplified Ras-MAPK signal states correlate with accelerated EGFR internalization, cytostasis and delayed HER2 tumor onset in Fer-deficient model systems. Oncogene.

[15] Zhong CY, Zhou YM, Douglas GC, Witschi H, Pinkerton KE (2005). MAPK/AP-1 signal pathway in tobacco smoke-induced cell proliferation and squamous metaplasia in the lungs of rats. Carcinogenesis.

[16] Zhao J, Harper R, Barchowsky A, Di YP (2007). Identification of multiple MAPK-mediated transcription factors regulated by tobacco smoke in airway epithelial cells. American journal of physiology Lung cellular and molecular physiology.

[17] Ebelt ND, Cantrell MA, Van Den Berg CL (2013). c-Jun N-Terminal Kinases Mediate a Wide Range of Targets in the Metastatic Cascade. Genes & cancer.

[18] Wei J, Li Z, Chen W, Ma C, Zhan F, Wu W, Peng Y (2013). AEG-1 participates in TGF-beta1-induced EMT through p38 MAPK activation. Cell biology international.

[19] Liao A, Wang W, Sun D, Jiang Y, Tian S, Li J, Yang X, Shi R (2015). Bone morphogenetic protein 2 mediates epithelial-mesenchymal transition via AKT and ERK signaling pathways in gastric cancer. Tumour biology.

[20] Mulholland DJ, Kobayashi N, Ruscetti M, Zhi A, Tran LM, Huang J, Gleave M, Wu H (2012). Pten loss and RAS/MAPK activation cooperate to promote EMT and metastasis initiated from prostate cancer stem/progenitor cells. Cancer research.

[21] Geng H, Zhao L, Liang Z, Zhang Z, Xie D, Bi L, Wang Y, Zhang T, Cheng L, Yu D, Zhong C (2015). ERK5 positively regulates cigarette smoke-induced urocystic epithelial-mesenchymal transition in SV40 immortalized human urothelial cells. Oncology reports.

[22] Pu J, Peng G, Li L, Na H, Liu Y, Liu P (2011). Palmitic acid acutely stimulates glucose uptake via activation of Akt and ERK1/2 in skeletal muscle cells. Journal of lipid research.

[23] Fang S, Jin Y, Zheng H, Yan J, Cui Y, Bi H, Jia H, Zhang H, Wang Y, Na L, Gao X, Zhou H (2011). High glucose condition upregulated Txnip expression level in rat mesangial cells through ROS/MEK/MAPK pathway. Molecular and cellular biochemistry.

[24] Ma J, Zhang L, Han W, Shen T, Ma C, Liu Y, Nie X, Liu M, Ran Y, Zhu D (2012). Activation of JNK/c-Jun is required for the proliferation, survival, and angiogenesis induced by EET in pulmonary artery endothelial cells. Journal of lipid research.

[25] Liang Z, Wu R, Xie W, Geng H, Zhao L, Xie C, Wu J, Geng S, Li X, Zhu M, Zhu W, Zhu J, Huang C, Ma X, Zhong C, Han H (2015). Curcumin Suppresses MAPK Pathways to Reverse Tobacco Smoke-induced Gastric Epithelial-Mesenchymal Transition in Mice. Phytotherapy research.

[26] Zhao L, Geng H, Liang ZF, Zhang ZQ, Zhang T, Yu DX, Zhong CY (2015). Benzidine induces epithelial-mesenchymal transition in human uroepithelial cells through ERK1/2 pathway. Biochemical and biophysical research communications.

[27] Friday BB, Adjei AA (2008). Advances in targeting the Ras/Raf/MEK/Erk mitogen-activated protein kinase cascade with MEK inhibitors for cancer therapy. Clinical cancer research.

[28] Saxena N, Lahiri SS, Hambarde S, Tripathi RP (2008). RAS: target for cancer therapy. Cancer investigation.

[29] Kuo PL, Chen YH, Chen TC, Shen KH, Hsu YL (2011). CXCL5/ENA78 increased cell migration and epithelial-to-mesenchymal transition of hormone-independent prostate cancer by early growth response-1/snail signaling pathway. Journal of cellular physiology.

[30] Zhang L, Chen W, Li X (2008). A novel anticancer effect of butein: inhibition of invasion through the ERK1/2 and NF-kappa B signaling pathways in bladder cancer cells. FEBS letters.

[31] Takeuchi A, Eto M, Shiota M, Tatsugami K, Yokomizo A, Kuroiwa K, Itsumi M, Naito S (2012). Sunitinib enhances antitumor effects against chemotherapy-resistant bladder cancer through suppression of ERK1/2 phosphorylation. International journal of oncology.

[32] Gong Y, Qiu W, Ning X, Yang X, Liu L, Wang Z, Lin J, Li X, Guo Y (2015). CCDC34 is up-regulated in bladder cancer and regulates bladder cancer cell proliferation, apoptosis and migration. Oncotarget.

[33] Kao YT, Hsu WC, Hu HT, Hsu SH, Lin CS, Chiu CC, Lu CY, Hour TC, Pu YS, Huang AM (2014). Involvement of p38 mitogen-activated protein kinase in acquired gemcitabine-resistant human urothelial carcinoma sublines. The Kaohsiung journal of medical sciences.

[34] Fang Y, Wang Y, Wang Y, Meng Y, Zhu J, Jin H, Li J, Zhang D, Yu Y, Wu XR, Huang C (2014). A new tumour suppression mechanism by p27Kip1: EGFR down-regulation mediated by JNK/c-Jun pathway inhibition. The Biochemical journal.

[35] Su Y, Han W, Giraldo C, De Li Y, Block ER (1998). Effect of cigarette smoke extract on nitric oxide synthase in pulmonary artery endothelial cells. American journal of respiratory cell and molecular biology.

[36] Tian D, Zhu M, Chen WS, Li JS, Wu RL, Wang X (2006). Role of glycogen synthase kinase 3 in squamous differentiation induced by cigarette smoke in porcine tracheobronchial epithelial cells. Food and chemical toxicology.

[37] Deng X, Zhong Y, Gu L, Shen W, Guo J (2013). MiR-21 involve in ERK-mediated upregulation of MMP9 in the rat hippocampus following cerebral ischemia. Brain research bulletin.

[38] Xiao J, Wang K, Feng YL, Chen XR, Xu D, Zhang MK (2011). Role of extracellular signal-regulated kinase 1/2 in cigarette smoke-induced mucus hypersecretion in a rat model. Chinese medical journal.

[39] Braem K, Luyten FP, Lories RJ (2012). Blocking p38 signalling inhibits chondrogenesis in vitro but not ankylosis in a model of ankylosing spondylitis in vivo. Annals of the rheumatic diseases.

[40] Ottani A, Galantucci M, Ardimento E, Neri L, Canalini F, Calevro A, Zaffe D, Novellino E, Grieco P, Giuliani D, Guarini S (2013). Modulation of the JAK/ERK/STAT signaling in melanocortin-induced inhibition of local and systemic responses to myocardial ischemia/reperfusion. Pharmacological research.

