# Supplementary material for: Cigarette smoke induced urocystic epithelial mesenchymal transition via MAPK pathways
Source: Oncotarget. 2017 Jan 2;8(5):8791–800. doi: 10.18632/oncotarget.14456 (PMC5352442; doi:10.18632/oncotarget.14456)
Supplement: Supplementary file 1 [file oncotarget-08-8791-s001.pdf]

Cigarette smoke induced urocystic epithelial mesenchymal transition via MAPK pathways

SUPPLEMENTARY METHODS

Chemicals and reagents

An SV-40 immortalized human urothelial cell line (SV-HUC-1) was purchased from Chinese Academy of Typical Culture Collection Cell Bank. F12K medium was purchased from Gibco (New York, NY, USA). Fetal bovine serum (FBS) was obtained from PAA Laboratories (Pasching, Austria). 3-(4,5-Dimethylthiazol-2-yl)-2,5-diphenyltetrazolium bromide (MTT) was purchased from Sigma-Aldrich. SB203580, SP600125, and U0126 were purchased from Beyotime (Shanghai, China). The primary antibodies for phosphorylated JNK, phosphorylated p38, phosphorylated ERK1/2, phosphorylated c-Jun, phosphorylated c-Fos, E-cadherin, ZO-1, N-cadherin and Vimentin were obtained from Cell Signaling Technology (Beverly, MA). GAPDH antibody was from Biogot Technology (Nanjing, China). Primers for E-cadherin, ZO-1, Vimentin, N-cadherin and GAPDH were synthesized by Invitrogen (Carlsbad, CA). Sources of other materials are noted accordingly in the text.

Cell culture

SV-HUC-1 cell lines was purchased from Chinese Academy of Typical Culture Collection Cell Bank (Shanghai, China). Cells were cultured in F12K medium (Gibco, Carlsbad, CA) supplemented with 10% fetal bovine serum (Gibco, Carlsbad, CA), penicillin (100U/ml) and streptomycin (100μg/ml) (Gibco, Carlsbad, CA). The cells were seeded in 10 cm^2 flasks. Cells cultures were maintained in a humidified incubator at 37°C with 5% CO_2. The medium was changed every other day until cells reached 80% confluence, and then treated with various concentrations of CS extract or with MAPK inhibitors.

Preparation of CSE

CSE was prepared daily immediately before use according to the reported method. Briefly, one filterless 3R4F reference cigarette (9 mg tar and 0.8 mg nicotine/cigarette) was combusted and the mainstream smoke was continuously drawn through a glass syringe containing 10 ml of fetal bovine serum-free F12K medium that was pre-warmed to 37°C at a rate of 5min/cigarette. The resulting suspension was adjusted to pH 7.4 and then filtered through a 0.22-μm-pore size filter. The obtained solution was referred to as a 100% CSE solution and further diluted to the desired concentration with culture medium. Control solution was prepared with the same protocol, except that the cigarette was unlit. SV-HUC-1 cells were exposed to various concentrations of CSE within 30 min of preparation.

Mice and CS exposure

Eight-week-old male BALB/c mice weighing 18-22 g were purchased from the Animal Research Center of Nanjing Medical University. All mice were allowed to acclimate for 1 week prior to the onset of experimental exposure. The mice were housed in polypropylene cages, maintained on a 12-hour light/dark cycle, 22±0.5°C room temperature, 40-60% relative humidity, and free access to water and AIN-76A diet. Animals were handled in accordance with the recommendations in the guidelines of the Animal Care and Welfare Committee of Nanjing Medical University. The study protocol was approved by the Committee on the Ethics of Animal Experiments of Nanjing Medical University.

Mice were exposed to cigarette smoke in a smoking apparatus. Filterless 3R4F reference cigarette was combusted to generate CS by a smoke machine, which smoked the cigarettes and pumped the mainstream cigarette smoke from burning cigarettes at a constant rate (each cigarette took 5 min to burn out). The smoke was delivered to whole-body exposure chambers with target concentration of total particulate matter (TPM) of 85 mg/m^3. Animals were exposed for 6 hours daily for 12 consecutive weeks. Animals in the control group were exposed to filtered, conditioned air. Ten mice were randomly assigned into each group. The exposures were monitored and characterized as the followings: carbon monoxide (14.72±2.89 mg/m^3), TSP (0 mg/m^3) for the control group; carbon monoxide (184.07±23.51 mg/m^3), TSP (83.53±5.63 mg/m^3) for CS exposure group. After the last CS exposure, mice were sacrificed and the bladder tissues were isolated, frozen and stored at -80°C until analysis.

Inhibitors of MAPK pathways treatment of mice

In a separate set of animal study, mice were treated daily with different MAPK inhibitors as previously reported [1–4]. All the inhibitors (0.5mg/kg for U0126, 1mg/kg for SB203580 and 1mg/kg for SP600125) were

dissolved in DMSO and intraperitoneally injected to the mice. mice were randomly assigned into six groups (n=10 per group): filtered air group; CS-exposed group, mice were exposed to CS; CS+DMSO group, mice were treated with equal volume DMSO and exposed to CS;CS+U0126 group, mice were treated with 0.5 mg/kg U0126 and exposed to CS; CS+SB203580 group, mice were treated with 1 mg/kg SB203580 and exposed to CS; CS+SP600125 group, mice were treated with 1 mg/kg SP600125 and exposed to CS; The intraperitoneal administration dose of inhibitors were based on the measurements of mouse body weight. Animals were weighed every three days. Mice were exposed to filtered air or CS with a target concentration of 85 mg/m^3 TPM for 12 weeks. Following the completion of exposure, mice were sacrificed and bladder tissues were collected for analysis. Examination of mouse survival, symptoms, body weight, diet consumption, hematology and blood biochemistry were also performed.

Cell toxicity assay

SV-HUC-1 cells were seeded in 96-well plates at a plating density of 2 $x10^3$ cells/well in 200 µl of medium. Then cells were exposed to various concentrations of CSE prepared as previously outlined for 5 days, and cell viability was determined by MTT assay. Five days later, MTT stock solution was added to each well to solubilize the formazan crystals, and plates were incubated for an additional 4 h at 37°C. Afterwards, MTT solution in the medium was removed and the crystals were solubilized in DMSO. Absorbance was measured at 490 nm using a microplate reader. All measurements were performed in triplicate.

Western blot analysis

Proteins were extracted from SV-HUC-1 cells and mouse bladder tissues. Protein concentrations were measured with the BCA Protein Assay (Beyotime Institute of Biotechnology, Shanghai, China). Afterwards, proteins were diluted to equal concentrations, boiled for 5 min and separated by 7.5-10% SDS–PAGE, followed by transbloting to a PVDF membrane (Millipore, Billerica, MA). The membranes were blocked with 5% defatted milk and subsequently probed with primary antibody overnight at 4°C. Horseradish peroxidase-conjugated secondary antibody was added for 1h at room temperature. GAPDH served as the loading control.

Quantitative real-time PCR

Total RNA was isolated by RNAiso Plus according to the manufacturer's instructions (TaKaRa, Japan). qRT-PCR was performed using Power SYBR Green Master Mix (TaKaRa, Japan) and an ABI 7300 real-time PCR detection system (Applied Biosystems). Fold changes in expression of each gene were calculated by a comparative threshold cycle (Ct) method using the formula $2^{-(\Delta\Delta Ct)}$. The primers used as follows: E-cadherin, forward 5′-TCGACACCCGATTCAAAGTGG-3′ and reverse 5′-TTCCAGAAACGGAGGCCTGAT-3′; ZO-1, forward 5′- GCAGCCACAACCAATTCATAG-3′ and reverse 5′-GCAGACGATGTTCATAGTTTC-3′; Vimentin, forward 5′-CCTTGACATTGAGATTGCCA-3′and reverse 5′-GTATCAACCAGAGGGAGTGA-3′; N-cadherin, forward 5′-ATCAAGTGCCATTAGCCAAG-3′ and reverse 5′- CTGAGCAGTGAATGTTGTCA -3′;GAPDH, forward 5′-GCTGCCCAACGCACCGAATA-3′ and reverse 5′- GAGTCAACGGATTTGGTCGT-3′.

Transwell assay

The invasion assays were performed in a 24-well Boyden chamber with an 8-µm pore size polycarbonate membrane (Millipore, Billerica, MA, USA) coated with Matrigel to form a matrix barrier. A total of 100 µl of serum-free medium (containing $1x10^4$ cells) was added to the upper compartment of the chamber, whereas the lower compartment was filled with 800 µl of F12K supplemented with 10% FBS. After incubation at 37°C for 48 h, the SV-HUC-1 cells remaining inside the upper chamber were removed with cotton swabs. The cells on the lower surface of the membrane were stained with 0.1% crystal violet after fixation with methanol and then imaged under a light microscope. Subsequently, the cells were bleached with glacial acetic acid and the absorbance of the eluant was measured at 570 nm.

Immunofluorescence

After CSE treatment for 5 days, SV-HUC-1 cells were fixed in 4% paraformaldehyde for 15 min at room temperature, and were washed three times with TBSTx. Then, membranes were ruptured with 0.1% TritonX-100 for 30 min. Slides were blocked in 5% BSA containing TBSTx for 1 h at room temperature, and then were incubated with a monoclonal E-cadherin (1:150 dilution) and Vimentin (1:100 dilution) antibodies in 5% BSA overnight at 4 C. After washing with TBSTx, cells were incubated with secondary antibodies for 1 h at room temperature. Finally, cells were counterstained with 4,60-diamidino-2-phenylindole (DAPI) before mounting, then cells were washed three times with TBSTx. Stained cells were mounted with fluorescent mounting medium (Dako, Carpinteria, CA, USA). The fluorescent images were obtained using a confocal laser scanning microscope (LSM700; Carl Zeiss Meditec, Gottingen, Germany).

REFERENCES

1. Deng, X., Zhong, Y., Gu, L., Shen, W., Guo, J., MiR-21 involve in ERK-mediated upregulation of MMP9 in the rat hippocampus following cerebral ischemia. Brain Res Bull, 2013. 94:p. 56-62.
2. Xiao, J., Wang, K., Feng, Y. L., Chen, X. R., Xu, D., Zhang, M. K., et al., Role of extracellular signal regulated kinase 1/2 in cigarette smoke-induced mucus hypersecretion in a rat model. Chin Med J (Engl), 2011. 124: p. 3327-33.
3. Braem, K., F.P. Luyten, R.J. Lories, Blocking p38 signalling inhibits chondrogenesis in vitro but not ankylosis in a model of ankylosing spondylitis in vivo. Ann Rheum Dis, 2012. 71: p. 722-8.
4. Ottani, A., Galantucci, M., Ardimento, E., Neri, L., Canalini, F., Calevro, A., et al., Modulation of the JAK/ERK/STAT signaling in melanocortin-induced inhibition of local and systemic responses to myocardial ischemia/reperfusion. Pharmacol Res, 2013. 72: p. 1-8.
